# Bioturbation and the δ^56^Fe signature of dissolved iron fluxes from marine sediments

**DOI:** 10.1098/rsos.220010

**Published:** 2023-01-25

**Authors:** Sebastiaan J. van de Velde, Andrew W. Dale, Sandra Arndt

**Affiliations:** ^1^ Department of Geoscience, Environment & Society, Université Libre de Bruxelles, Av. F. Roosevelt 50, CP160/02, 1050 Brussels, Belgium; ^2^ Operational Directorate Natural Environment, Royal Belgian Institute of Natural Sciences, Rue Vautier 29, Brussels, Belgium; ^3^ GEOMAR Helmholtz Centre for Ocean Research Kiel, Wischhofstr. 1-3, D-24148 Kiel, Germany

**Keywords:** benthic iron flux, iron isotopes, bioturbation, diagenetic modelling

## Abstract

We developed a reaction-transport model capable of tracing iron isotopes in marine sediments to quantify the influence of bioturbation on the isotopic signature of the benthic dissolved (DFe) flux. By fitting the model to published data from marine sediments, we calibrated effective overall fractionation factors for iron reduction (–1.3‰), oxidation (+0.4‰), iron-sulfide precipitation (+0.5‰) and dissolution (−0.5‰) and pyrite precipitation (−0.7‰) that agree with literature values. Results show that for bottom-water oxygen concentrations greater than 50 µM, higher bioturbation increased the benthic DFe flux and its *δ*^56^Fe signature. By contrast, for oxygen concentrations less than 50 µM, higher bioturbation decreased the benthic DFe flux and its *δ*^56^Fe signature. The expressed overall fractionation of the benthic DFe flux relative to the *δ*^56^Fe of the iron oxides entering the sediment ranges from −1.67‰ to 0.0‰. On a global scale, the presence of bioturbation increases sedimentary DFe release from approximately 70 G mol DFe yr^−1^ to approximately 160 G mol DFe yr^−1^ and decreases the *δ*^56^Fe signature of the DFe flux.

## 1. Introduction

Iron plays a central role in marine biogeochemical cycles. Over the last 100 000 years, iron has been a limiting micronutrient for marine primary productivity in large parts of the ocean and may have been a driver for glacial–interglacial cycles by modulating atmospheric CO_2_ concentrations [1,2]. Understandably, much work in the past decades has focused on understanding and modelling the oceanic iron cycle [3–5]. Yet, many of its aspects remain poorly constrained, mainly owing to our incomplete understanding of dissolution and scavenging processes [6,7], as well as the difficulty of quantifying iron supply from hydrothermal and other sediment sources [8–12]. Continental shelf and slope sediments in particular are recognized as important contributors to the global iron budget. Sediments can release iron to the bottom water as reduced dissolved ferrous iron (DFe) [10,13] or as particulate iron (oxyhydr)oxides (FeOOH; mainly represented by goethite, ferrihydrite and haematite; from hereon referred to as iron oxides) by resuspension of the oxidized surface layer [8]. The potential DFe flux from continental shelves and slopes is estimated to be approximately 100 G mol yr^−1^ (excluding sources from hydrothermal vents) [9], while the global significance of the resuspended particulate flux is currently unknown. The benthic DFe flux, therefore, exceeds the estimated DFe delivery via rivers (approx. 2.5 G mol yr^−1^; [14]), glaciers (approx. 0.04 G mol yr^−1^; [15]), hydrothermal vents (approx. 0.9 G mol yr^−1^; [16]) and dust deposition (1–33 G mol yr^−1^; [5]). Continental shelf and slope sediments are thus potentially the major source of DFe to the ocean.

The magnitude of the benthic (non-hydrothermal) iron source is modulated by both the amount and reactivity of FeOOH settling on the sediment surface, the organic carbon mineralization rate in the sediment and bottom-water oxygen concentrations [9,10,17–20]. Benthic DFe release is positively correlated with the organic carbon mineralization rate in the sediment through coupling with dissimilatory reduction of FeOOH [10,17]. By contrast, bottom-water oxygen concentration negatively correlates with benthic DFe flux [13,18] as a result of reoxidation of DFe to FeOOH [9]. If bottom waters turn anoxic and sulfidic, DFe fluxes may eventually decrease due to the formation of reduced iron sulfide minerals [18–20]. These biogeochemical controls have recently been quantitatively evaluated on a global scale in a diagenetic model study [9]. Results showed that the benthic DFe flux (*J*_DFe_, units are µmol m^−2^ d^−1^) can be expressed as a function of the FeOOH rain rate (*J*_FeOOH,T_ , units are µmol m^−2^ d^−1^), the sedimentary organic carbon mineralization rate (Cox, units are mmol m^−2^ d^−1^), and bottom-water oxygen concentrations ([O_2_]_BW_, units are µM). Based on model results, a transfer function for DFe fluxes was derived [9],

1.1 $$J_{\mathrm{DFe}}=0.153J_{\mathrm{FeOOH},T}^{\;}\tanh\left(\frac{\mathrm{Cox}}{{\left[O_{2}\right]}_{\mathrm{BW}}}\right).$$

Note that in [9], *J*_FeOOH,T_ was assumed to be 1110 µmol m^−2^ d^−1^, so that 0.153 *J*_FeOOH,T_ = 170 µmol m^−2^ d^−1^, which was defined as the maximum potential DFe flux away from river mouths. In equation (1.1), bioturbation is not explicitly included but is assumed to be dependent on [O_2_]_BW_, and hence its potential impact on DFe fluxes has not been directly assessed [9]. However, field observations from estuarine, coastal and shelf sediments have shown that bioturbation exerts an important control on sediment–water DFe fluxes [10,13,17,21].

The term bioturbation comprises a variety of animal behaviours, which are typically grouped into two categories; ‘bio-irrigation’, which describes the transport of dissolved species through animal burrows, and ‘bio-mixing’, which describes the transport of solid-phase particles [22,23]. Both these aspects of bioturbation have contrasting effects on the sedimentary Fe biogeochemistry [24]. Bio-irrigation increases the solute exchange between sediment and water column [22,24,25] and local studies in coastal and estuarine sediments suggest that bio-irrigation increases the benthic DFe flux [21,26]. Bio-mixing, on the other hand, stimulates Fe cycling within the sediment column [24,27–30] and its influence on benthic recycling fluxes tends to be highly dependent on the redox zonation and thus on the complex and dynamic network of biogeochemical processes in marine sediments [29,31]. The role of bio-irrigation and bio-mixing in modulating benthic DFe fluxes on the global scale is largely unknown.

Additionally, no global assessment of the isotopic signatures of benthic DFe fluxes, analogous to equation (1.1), exists. However, such quantification would be a particularly powerful tool to better constrain marine iron sources and sinks in past and present oceans [32,33]. Iron has four stable isotopes (^54^Fe, ^56^Fe, ^57^Fe and ^58^Fe), of which ^56^Fe and ^54^Fe are the most abundant. Accordingly, the ^56^Fe isotopic signature, calculated as the deviation in ‰ of the ^56^Fe/^54^Fe ratio relative to the IRMM−014 reference standard (*δ*^56^Fe; [34]), is commonly used to constrain the individual sources or sinks of Fe in the ocean (e.g. [35]). For example, particulate iron delivered to the oceans via aerosol deposition or riverine discharge at low latitudes has a *δ*^56^Fe signature of approximately 0.0‰ [36]. Dissolved Fe that is released from continental shelves and slopes after reduction of particulate FeOOH in the sediment generally has a low *δ*^56^Fe signature of approximately −2.0‰ [13,37], whereas iron released following non-reductive dissolution in passive margins has a *δ*^56^Fe of approximately 0.0‰ [38,39]. Currently, however, assessments of the isotopic signature of DFe released from the sediment are scarce. Consequently, our understanding of its response to different environmental conditions, as well as of the *δ*^56^Fe signature of the benthic iron source at the global scale is poorly constrained, limiting the accuracy of ocean biogeochemical models [12].

Here, we address this gap by extending the work of [9] and tackling two major uncertainties in the marine iron cycle: (i) the importance of bioturbation for the global benthic DFe flux, and (ii) the *δ*^56^Fe signature of the global benthic DFe flux. First, we combine reaction-transport modelling with previously published field data to determine effective overall iron isotope fractionation factors for the most important Fe diagenetic reactions in our model. Note that these are not equivalent to equilibrium or kinetic isotope fractionation factors as derived from laboratory experiments, but should be considered as apparent fractionation factors in marine shelf sediments. We then extend a previously published and validated diagenetic model [9] to track iron isotopes and use this model to investigate the effect of bioturbation, idealized as biodiffusive and non-local transport, on the benthic DFe flux and its isotopic signature under a range of different bottom-water redox conditions. Finally, we derive two sets of predictive global functions for the magnitude and isotopic signature of the benthic DFe flux based on benthic carbon oxidation rates, bottom-water oxygen concentrations and iron oxide rain rates; (i) for the modern seafloor, and (ii) for an unbioturbated seafloor akin to the Precambrian seafloor. Ultimately, this work contributes to the improvement of the predictive capacity of global ocean biogeochemical models.

## 2. Material and methods

### 2.1. Approach

Our approach consisted of two separate steps, for which we designed two different diagenetic models. First, we calibrated overall effective iron isotope fractionation factors for the most pertinent biogeochemical reactions by combining available field data with a site-specific one-dimensional reaction-transport model of marine sediments. The model was applied to two field sites for which solid-phase and pore-water iron concentrations and their isotope values were available (‘site-specific model’; electronic supplementary material, Info. §1). Due to the lack of a complete set of field data at the two field sites, the model set-up for the two case studies did not explicitly resolve nitrogen and manganese cycles. Biogeochemical reactions in marine sediments are complex and can involve multiple steps (e.g. the oxidation-precipitation reaction from Fe^2+^ to FeOOH probably proceeds through the ligand-bound Fe^3+^ intermediate [40]). In most reaction-transport models—as in the ones used here—all these intermediate reaction steps are lumped in one reaction, the rate of which is controlled by the slowest step. Each of these intermediate reactions can induce different kinetic or equilibrium isotope fractionations, and the sum of the fractionations for the overall reaction can be described by an apparent or ‘effective’ fractionation factor (*α*_eff_) for the overall reaction [40]. In reality, the magnitude of *α*_eff_ will be dependent on the reaction rate. Currently, however, very little is known about the relation between the reaction rate and *α*_eff_ for the most important sedimentary biogeochemical iron reactions. For this reason, we only derived a single *α*_eff_ for each of the modelled reactions. Despite this assumption, we show below that we can derive acceptable model–data fits for the two field sites with very contrasting regimes of iron cycling, which suggests that our model can provide information on iron cycling more widely. Nevertheless, future developments should target more realistic descriptions of iron fractionation in marine sediments.

In the second step, we extended the diagenetic model used in [9] to track iron isotope signatures using the effective iron isotope fractionation factors constrained in step 1 (‘idealized model’; electronic supplementary material, Info. §2). The model set-up of [9] has been previously validated against a global database of benthic iron fluxes, and explicitly accounts for the major known network of biogeochemical reactions observed in global marine sediments, including nitrogen and manganese cycling. We used the idealized model set-up in a global sensitivity analysis aimed at assessing the importance of bioturbation and deriving predictive functions linking benthic DFe fluxes and their isotopic signature to their main environmental controls (i.e. Cox, [O_2_]_BW_ and J_FeOOH,T_) for both modern bioturbated marine sediments and unbioturbated sediments. These predictive functions were subsequently used to quantify the importance of bioturbation for the global benthic DFe flux.

### 2.2. Model description

We used two vertically resolved one-dimensional reaction-transport models to simulate the coupled biogeochemical cycles of C, O, N, Mn, Fe and S (C, O, Fe and S in the case of the site-specific model). The two models only differed in implemented reaction network and boundary conditions. Solid transport occurs via sediment accumulation and bio-mixing. Solutes are transported by molecular diffusion and bio-irrigation. Bio-mixing is implemented as a diffusion-like process [41], whereas bio-irrigation is described as a non-local exchange process [42]. The depth-dependent advection velocities of solids and solutes is calculated from the porosity profile and the burial velocities in compacted sediments. The model formulation is informed by previous empirical models [9,24,30,43–46]. Electronic supplementary material, Info. §§1 and 2 provide detailed descriptions of the two diagenetic model set-ups. Here, we only briefly discuss the implemented Fe cycle.

**Figure 1.**
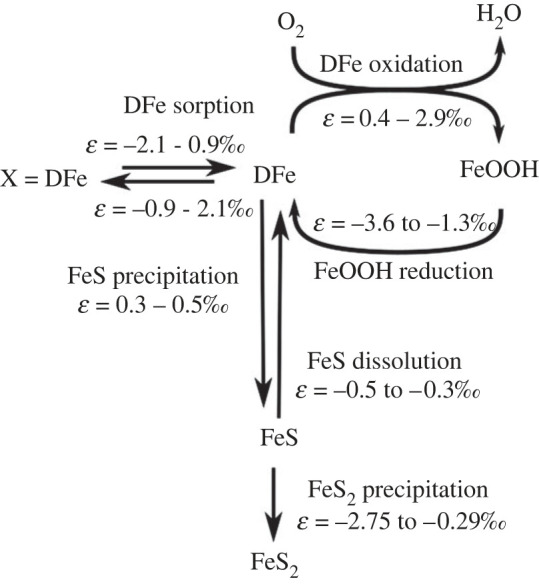
Simplified iron cycle in marine sediments. In the model, iron oxides (FeOOH) are modelled as four separate fractions, defined on their reactivity toward sulfide. FeOOH reduction can be coupled to organic matter oxidation (only the HR fraction) or sulfide oxidation (all fractions). Isotope fractionation factors (*ε*) are taken from the literature, and comprise both equilibrium and kinetic effects [47–60].

The Fe cycle in the idealized model (figure 1) explicitly accounts for four particulate iron oxide fractions that are defined by their reactivity according to wet chemical extraction methods [61–64]. The half-lives of the different iron fractions are defined relative to the reaction with sulfide [65]. The most reactive fraction (‘highly reactive’, FeHR) includes amorphous and reactive crystalline oxides and has a half-life of less than 1 year. The second most reactive fraction ‘moderately reactive’ Fe (FeMR) represents more crystalline oxides such as goethite and magnetite, as well as reactive silicates, and has a half-life of approximately 100 years [63]. The ‘poorly reactive’ Fe (FePR) fraction encompasses mostly reactive silicates with a half-life of approximately 100 000 years. The ‘unreactive’ iron (FeU) fraction includes Fe bound within silicates that do not react on timescales relevant to this study. The reduction of iron oxides releases DFe to the pore-water, which can then adsorb on solid-phase particles, be reoxidized to FeHR, or precipitate as iron mono-sulfide (FeS). FeS can be further transformed to pyrite (FeS_2_) by reaction with dissolved sulfide or elemental sulfur. More reactive iron classes can age into less reactive fractions. For the site-specific model, we omitted moderately reactive, poorly reactive and unreactive iron mineral classes because of the lack of empirical information with respect to the less reactive iron classes. We did, however, allow the highly reactive class to comprise ‘fresh’ and ‘aged’ iron oxides, following previous studies [30,45]. Note that in both model set-ups, we did not include non-reductive dissolution of Fe minerals, which is potentially important in sediments characterized by low rates of organic matter mineralization [38,39]. Non-reductive dissolution is mechanistically not well understood, making it difficult to include it in diagenetic models. However, because benthic DFe fluxes driven by this dissolution process are very low (approx. 1 µmol m^−2^ d^−1^) [38,39], it is probably of minor importance for our study.

In addition to total (bulk) Fe, the implemented Fe cycle also tracks the ^56^Fe concentration of all Fe species. For simplicity, we assumed that the bulk fraction only consisted of the two major Fe isotopes; ^54^Fe and ^56^Fe (which constitute greater than 97% of the total iron pool). Accordingly, the *δ*^56^Fe value in dissolved Fe species is calculated as

2.1 $$\delta^{56}{\mathrm{Fe}}_{C_{i}}=\left(\frac{\left({\;}^{56}C_{i}/(C_{i}{-}^{56}C_{i})\right)}{{\left({\;}^{56}\mathrm{Fe}{/}^{54}\mathrm{Fe}\right)}_{\mathrm{ref}}}-1\right).1000,$$

where *C_i_* represents the concentration of bulk Fe in Fe species *i*, ^56^*C_i_* is the concentration of ^56^Fe in Fe species *i* and ${\left({\;}^{56}\mathrm{Fe}{/}^{54}\mathrm{Fe}\right)}_{\mathrm{ref}}$ is the isotope ratio of a standard sample (15.697861 for IRMM-14; [34]). Each reaction *R*_k_ (which tracks the reaction of the bulk species) has a corresponding isotope-specific reaction ^56^*R*_k_ that is related to *R*_k_ by the effective fractionation factor for the overall reaction ${\;}^{56}\alpha_{\mathrm{eff},R_{k}}$ [40]

2.2 $${\;}^{56}R_{k}=\frac{{\;}^{56}\alpha_{\mathrm{eff},R_{k}}.^{56}r_{C_{i}}}{1+{\;}^{56}\alpha_{\mathrm{eff},R_{k}}.^{56}r_{C_{i}}}R_{k},$$

where ${\;}^{56}r_{C_{i}}$ represents the ^56^Fe/^54^Fe isotope ratio of *C_i_*,

2.3 $${\;}^{56}r_{C_{i}}=\frac{{\;}^{56}C_{i}}{C_{i}-{\;}^{56}C_{i}}.$$

In this study, the effective fractionation factor ${\;}^{56}\alpha_{\mathrm{eff},R_{k}}$ is defined as greater than 1 when the reaction fractionates toward more positive isotopes (the remaining Fe pool becomes more negative), and less than 1 when the reaction fractionates towards more negative isotopes (the remaining Fe pool becomes more positive). For ease of notation, we report the effective fractionation factor ${\;}^{56}\alpha_{\mathrm{eff},R_{k}}$ in the epsilon-notation (${\;}^{56}\varepsilon_{\mathrm{eff},R_{k}}$; expressed in ‰)

2.4 $${\;}^{56}\varepsilon_{\mathrm{eff},R_{k}}=1000{(}^{56}\alpha_{\mathrm{eff},R_{k}}-1).$$

To avoid extreme *δ*^56^Fe values at low bulk concentrations, a fractionation limit (*C*_lim_) was set at 10^−9^ µmol cm^−3^ of sediment. Reactions that proceeded below this bulk concentration induce no isotope fractionation,

2.5 $${\;}^{56}R_{k}[C_{i}<C_{\lim}]=\frac{{\;}^{56}C_{i}}{C_{i}}R_{k}.$$

Adsorption of ferrous iron to clay or oxide minerals [65] is modelled as an instantaneous reversible equilibrium [45],

2.6 $$[X\equiv {\mathrm{Fe}}^{2+}]=K_{\mathrm{ads}}^{{\mathrm{Fe}}^{2+}}[{\mathrm{Fe}}^{2+}],$$

where $K_{\mathrm{ads}}^{{\mathrm{Fe}}^{2+}}$ is a dimensionless adsorption constant [45]. To account for effective isotope fractionation during adsorption, the pool of adsorbed ^56^Fe is calculated as

2.7 $$[X\equiv {\;}^{56}{\mathrm{Fe}}^{2+}]=K_{\mathrm{ads}}^{{\mathrm{Fe}}^{2+}}\frac{{\;}^{56}\alpha_{\mathrm{eff},\mathrm{FIS}}+{\;}^{56}\alpha_{\mathrm{eff},\mathrm{FIS}}.^{56}r_{{\mathrm{Fe}}^{2+}}}{1+{\;}^{56}\alpha_{\mathrm{eff},\mathrm{FIS}}.^{56}r_{{\mathrm{Fe}}^{2+}}}{[}^{56}{\mathrm{Fe}}^{2+}],$$

where ${\;}^{56}\alpha_{\mathrm{eff},\mathrm{FIS}}$ is the effective fractionation factor associated with ferrous iron sorption, and all other parameters have been defined previously. The model is implemented in the open-source programming language R [47], following the procedures of [48]. The reader is referred to the electronic supplementary material for further information about the model implementation, parametrization (tables 1 and 2) and solution.

**Table 1.** List of boundary conditions and parameters used in the reaction-transport model used for calibration of the effective isotope fractionation factors. Solid-phase and pore-water concentrations are expressed per unit volume of solid phase and pore water, respectively. ‘method’ refers to the procedure by which parameter values are constrained: A = Literature values, B = model calibration. Note that all isotope values are given relative to the IRMM-14 standard. MC = Monterey Canyon, SBB = Santa Barbara Basin.

	symbol	value		units	method	references
		SBB	MC			
environmental parameters						
temperature	T	10	10	°C	A	[46,53]
salinity	S	34.2	34.2	—	A	[46,53]
porosity (surface value)	$\phi_{F}^{0}$	0.95	0.95	—	A	[46,53]
porosity (asymptotic at depth)	$\phi_{F}^{\infty }$	0.82	0.82	—	A	[46,53]
porosity attenuation coefficient	$x_{\phi }$	3.6	3.6	cm	A	[46,53]
solid-phase density	*ρ_S_*	2.6	2.6	g cm^−3^	A	[46,53]
burial velocity in compacted sediment	*v_S_*, *v*_F_	250	250	cm kyr^−1^	A	[54,55]
bio-mixing depth	*z_L_*	0	10	cm	B	
biodiffusion coefficient	*D_b_*_,0_	0	20	cm^2^ yr^−1^	B	
bio-irrigation coefficient	*α*_0_	0	183	yr^−1^	B	
bio-irrigation attenuation coefficient	*x*_irr_	0	3	cm	B	
depth of sediment domain	L	150	150	Cm	—	
^56^Fe/^54^Fe isotope ratio of IRMM014	—	15.697861		—	A	[34]
boundary conditions						
oxygen bottom water	[O_2_]	0.01	0.28	mol m^−3^	A	[13,53,56]
sulfate bottom water	$\left[{\mathrm{SO}}_{4}^{2-}\right]$	28.0	28.0	mol m^−3^	A	[13,53,56]
DIC bottom water	∑CO_2_	2.45	2.45	mol m^−3^	A	[46,53]
ferrous iron bottom water	[DFe]	0	0	mol m^−3^	A	[46,53]
free sulfide bottom water	[HS^−^]	0	0	mol m^−3^	A	[46,53]
methane bottom water	[CH_4_]	0	0	mol m^−3^	A	[46,53]
flux particulate organic carbon	*J*_POC_	4.6	8.0	mmol m^−2^ d^−1^	B	
flux FeOOH_T_	*J*_FeOOH,T_	0.56	0.32	mmol m^−2^ d^−1^	B	
isotopic signature of FeOOH_T_	*δ*^56^Fe_FeOOH_	−1.5	−0.5	‰	B	
flux FeS	*J*_FeS_	0	0	mmol m^−2^ d^−1^	B	
isotopic signature of FeS	*δ*^56^Fe_FeS_	—	—	‰	B	
flux FeS_2_	*J*_FeS2_	0.03	0.03	mmol m^−2^ d^−1^	B	
isotopic signature of FeS_2_	*δ*^56^Fe_FeS2_	−0.4	0.0	‰	B

**Table 2.** Boundary conditions for the baseline simulation (idealized model). Invariable parameters across all simulations are given in the electronic supplementary material. All isotope values are given relative to the IRMM-14 standard.

boundary conditions	symbol	baseline value	sensitivity analysis	units
characteristic water depth^a^	—	350	350	m
temperature^b^	T	10	10	°C
sediment accumulation rate at infinite depth^c^	$v_{s},v_{F}$	60	60	cm kyr^−1^
oxygen bottom water	[O_2_]_BW_	120	1–200^d^	µM
sulfate bottom water	${\left[{\mathrm{SO}}_{4}^{2-}\right]}_{\mathrm{BW}}$	28	0–28^e^	mM
ferrous iron bottom water	[DFe]_BW_	0	0	µM
isotopic signature	*δ*^56^Fe _DFe_	—	—	‰
POC rain rate^f^	*J*_POC_	10	0.5–16^g^	mmol m^−2^ d^−1^
flux FeOOH_T_^h^	*J*_FeOOH,T_	1110	194–1914^i^	µmol m^−2^ d^−1^
isotopic signature of FeOOH_T_	*δ*^56^Fe_FeOOH,T_	0.0	0.0	‰
flux FeS	*J*_FeS_	0.0	0.0	µmol m^−2^ d^−1^
isotopic signature of FeS	*δ*^56^Fe_FeS_	—	-	‰
flux FeS_2_	*J*_FeS2_	0.0	0.0	µmol m^−2^ d^−1^
isotopic signature of FeS_2_	*δ*^56^Fe_FeS2_	—	—	‰
bioturbation parameters				
bio-diffusion coefficient^j^^,^^k^	*D_b_*_,0_	10.*f*	variable	cm^2^ yr^−1^
mixing depth^l^	*z*_L_	$z_{L}=1.0+9.0\times (1-e^{-D_{b,0}/30})$	variable	cm
bio-irrigation coefficient^j,^^m^^,^^n^	*α*_0_	290.*f*	variable	yr^−1^

^a^[49].

^b^[50].

^c^[51].

^d^Tested values were 1, 2, 5, 10, 15, 25, 50, 100, 120 and 200 µM.

^e^Only tested for the ‘unbioturbated seafloor’ experiment. Tested values were 0, 0.01, 0.1, 1 and 28 mM.

^f^Estimated mean carbon oxidation rate for sediments less than 200 m water depth by [52].

^g^Tested values were 0.5, 1, 2, 4, 6, 8, 10, 12, 14 and 16 mmol C m^−2^ d^−1^, which gives carbon oxidation rates of 0.4, 0.8, 1.6, 3.3, 4.9, 6.6, 8.3, 9.9, 11.6 and 13.2 mmol C m^−2^ d^−1^ (the difference is due to particulate organic carbon (POC) burial below the model domain).

^h^Flux value of total iron oxides for the standard model of [9], 50% of this flux is considered unreactive [66], and the other 50% is divided equally among FeHR, FeMR and FePR [9].

^i^Tested values were: 194, 278, 555, 1110 and 1914 µmol m^−2^ d^−1^.

^j^Mean bio-diffusion coefficient of the modern data compilation of [67].

^k^*f* represents a dimensionless factor that scales bio-mixing and bio-irrigation coefficients to bottom-water oxygen (as introduced by [9]). *f* equals 0.5 + 0.5_erf_ (([O_2_]_BW_ − *a*)/*b*) where *a* = 20 µM and *b* = 12 µM [9].

^l^Mixing depth is calculated from the bio-diffusion coefficient as $z_{L}=1.0+9.0\times (1-e^{-D_{b,0}/30})$ as introduced previously by [24] (see electronic supplementary material).

^m^Following [9,68], the solute-specific Fe^2+^ bio-irrigation parameter is 20% of the bio-irrigation coefficient, and the solute-specific HS^−^ bio-irrigation coefficient is 50% of the bio-irrigation coefficient.

^n^The attenuation coefficient of bio-irrigation is kept constant at 1.4 cm during the sensitivity analysis.

### 2.3. Calibration of effective overall isotopic fractionation factors

The site-specific model set-up used here resolves the biogeochemical cycling of all chemical species that can be constrained by field data (i.e. FeOOH, FeS, FeS_2_ and DFe) in the upper 150 cm of the sediment column. To derive best-fit effective isotopic fractionation factors, we used datasets from sites in Monterey Canyon and Santa Barbara Basin [54]. The datasets include concentrations of pore-water Fe and Fe-bearing minerals and their respective *δ*^56^Fe values. They cover two contrasting sites; a bioturbated site underlying a fully oxygenated water column (Monterey Canyon), and an unbioturbated site underlying a hypoxic (less than 10 µM O_2_) water column (Santa Barbara Basin) (table 1; [54]). We first fitted the bulk concentrations of dissolved Fe (DFe), HCl-extractable Fe (FeHCl) (which includes FeOOH, sorbed Fe^2+^ and FeS) and pyrite (FeS_2_). Subsequently, the effective isotope fractionations were determined by finding the best model–data fit to the *δ*^56^Fe signature of the three distinct Fe pools. Site-specific boundary conditions were constrained based on observational data and are provided in table 1.

### 2.4. Model sensitivity experiments: assessing the role of bioturbation and derivation of predictive functions

All sensitivity experiments described below were performed using the idealized model. A detailed description of the set-up is provided in electronic supplementary material, Info. §2. The boundary conditions and bioturbation parameters of the baseline simulation were chosen to represent an idealized shelf sediment, and all parameter values were selected from compiled datasets or previously published studies (table 2; following [9]). Effective fractionation factors for each iron reaction were based on the derived effective isotope fractionation factors from our local case studies and compared with literature values (§3.1 and table 4).

**Table 4.** Modelled effective fractionation factors compared with *in situ* and laboratory values reported in the literature.

reaction	Reactant	product	effective fractionation factor $\left({\;}^{56}\varepsilon_{\mathrm{eff},R_{k}}\right)$		references
			model	literature range	
dissimilatory iron reduction^a^	FeOOH	DFe	−1.3‰	−3.6‰ to −1.3‰	[36,72–75]
ferrous iron oxidation^b^	DFe/FeS/FeS_2_	FeOOH	+0.4‰	+0.4‰ to +2.9‰	[76–78]
ferrous iron adsorption	DFe	X = DFe	+0.4‰	−0.9‰ to +2.1‰	[73,79,80]
iron sulfide precipitation	DFe	FeS	+0.5‰	+0.3‰ to +0.5‰	[81,82]
iron sulfide dissolution	FeS	DFe	−0.5‰	−0.5‰ to −0.3‰	[81,82]
pyrite precipitation	FeS	FeS_2_	−0.4‰ (MC) −0.7‰ (SBB)	−2.75‰ to −0.29‰	[76,83,84]

^a^Dissimilatory iron reduction coupled to organic matter mineralisation and sulphide oxidation are assigned the same fractionation factor.

^b^All oxidation reactions (i.e. iron sulphide oxidation and pyrite oxidation) are assigned the same fractionation factor.

Five model sensitivity experiments were designed to investigate the effect of bioturbation on the magnitude and isotopic signature of the benthic DFe flux:

− ‘Baseline’: both bio-mixing and bio-irrigation are dependent on bottom-water oxygen concentrations (table 2).
− ‘Unbioturbated’: bio-mixing and bio-irrigation parameters are set to zero.
− ‘Always bioturbated’: bio-mixing and bio-irrigation parameters are set to their maximum value (*D*_b,0_ = 10 cm^2^ yr^−1^, *z*_L_ = 9.7 cm, *α*_0_ = 290 yr^−1^; table 2) and independent of oxygen concentrations.
− ‘Only bio-mixing’: bio-mixing parameters are set at their maximum value (*D*_b,0_ = 10 cm^2^ yr^−1^, *z*_L_ = 9.7 cm; table 2) and independent of bottom-water oxygen concentrations. Bio-irrigation parameters are set to zero.
− ‘Only bio-irrigation’: bio-mixing parameters are set to zero. Bio-irrigation parameters are set at their maximum value (*α*_0_ = 290 yr^−1^; table 2) and independent of bottom-water oxygen concentrations.

For each of the five experiments listed above, bottom-water oxygen concentrations are varied between 1 and 200 µM. The remaining boundary conditions are set to their baseline values (table 2).

In addition, we ran a further two sets of extended sensitivity experiments to derive predictive functions for the magnitude and isotopic signature of the benthic DFe flux as a function of Cox, [O_2_]_BW_ and *J*_FeOOH,T_:

− ‘Modern seafloor’: bio-mixing and bio-irrigation parameters are dependent on bottom-water oxygen concentrations based on the relationship proposed by [9] (table 2). Particulate organic carbon (POC) rain rate (*J*_POC_, which determines Cox – table 2) and bottom-water oxygen concentrations ([O_2_]_BW_) are varied across the range typically observed in shelf and slope bottom waters, i.e. 0.5 and 16 mmol C m^−2^ d^−1^ and 1 and 200 µM O_2_, respectively (table 2). We carried out this sensitivity experiment for a range of plausible total FeOOH (FeOOH_T_) fluxes (194 to 1914 µmol Fe m^−2^ d^−1^; table 2).
− ‘Unbioturbated seafloor’: bio-mixing and bio-irrigation parameters are set to zero, and we tested the same ranges of environmental conditions described above. In addition to varying total FeOOH fluxes, we also ran the model over a range of sulfate concentrations between 0 and 28 mM to test the potential influence of lower sulfate concentrations, as observed during the Quaternary [57].

We did not explicitly test the influence of changes in organic matter (OM) reactivity on DFe fluxes and their isotopic signature. The model uses a fixed reactivity distribution for organic matter which is representative of a 2-year-long fresh phytoplankton decay experiment [58]. This parametrization thus overestimates OM reactivity in depositional settings that receive large loads of less reactive terrestrial, physically protected and/or pre-aged OM. We also do not take into account any potential effects of bioturbation or anoxic conditions on the degradation of organic matter [30,59]. Because the controls on organic matter reactivity in sediments are still a matter of extensive debate (see e.g. [60]), and outside the scope of this paper, we chose to keep organic matter reactivity fixed for the idealized model runs.

### 2.5. Quantifying the importance of bioturbation for the global benthic DFe flux

The predictive functions were subsequently used to derive a global estimate of the benthic DFe flux and its *δ*^56^Fe signature for the modern seafloor and a seafloor without any bioturbation. We used [O_2_]_BW_ from World Ocean Atlas 2018 on a 1 × 1 resolution (available at https://www.nodc.noaa.gov/OC5/woa18/). We then combined this with estimated Cox rates for each of the bathymetric intervals [52]. We calculated the mean and total DFe flux (*J*_DFe_) for several water depth intervals, as well as the mean *δ*^56^Fe signature of the DFe flux. Because no information about the spatial distribution of FeOOH fluxes is currently available, we assumed a globally uniform *J*_FeOOH,T_ of 1110 µmol m^−2^ d^−1^, to be consistent with previous work [9]. However, in reality, the deposition of FeOOH is not uniform but varies geographically [69]. This choice mainly affects the estimated global flux, and does not greatly alter our conclusions on the relative impact of bioturbation on sedimentary Fe release and isotope dynamics (as these are independent of the FeOOH influx; see below). This issue could be addressed in the future by coupling the proposed benthic Fe flux equations to a pelagic Fe model (such as cGEnIE.muffin, PISCES or UVic; [32,70,71]), which would greatly improve global benthic Fe flux predictions.

## 3. Results and discussion

### 3.1. Determination of effective iron isotope fractionation factors

**Figure 2.**
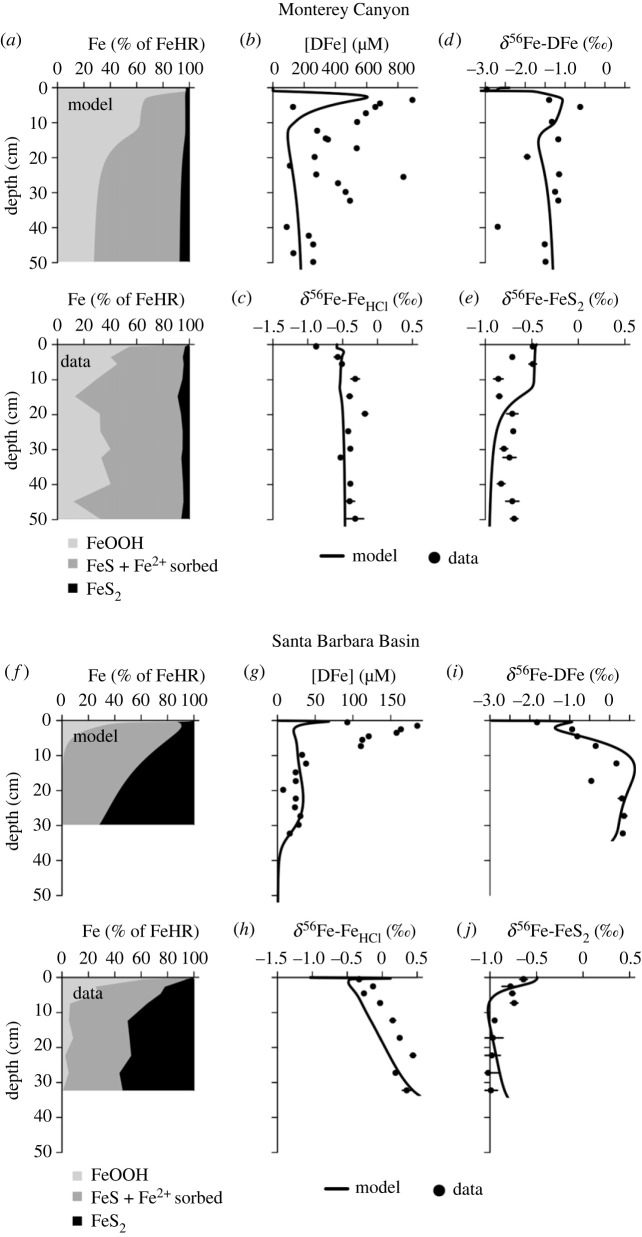
Model fit to the data from Monterey Canyon and Santa Barbara Basin [54]. Monterey Canyon: (*a*) modelled and measured fractions of highly reactive iron minerals (FeHR). (*b*) Dissolved Fe (DFe) concentrations. *δ*^56^Fe of (*c*) HCl-extractable Fe minerals (*δ*^56^Fe-Fe_HCl_), which includes FeOOH, sorbed Fe^2+^ and FeS, (*d*) dissolved Fe (*δ*^56^Fe-DFe), (*e*) pyrite (*δ*^56^Fe-FeS_2_). Santa Barbara Basin: (*f*) modelled and measured fractions of highly reactive iron minerals (FeHR). (*g*) Dissolved Fe (DFe) concentrations. *δ*^56^Fe compositions of (*h*) HCl-extractable Fe minerals (*δ*^56^Fe-Fe_HCl_), (*i*) dissolved Fe (*δ*^56^Fe-DFe), (*j*) pyrite (*δ*^56^Fe-FeS_2_). Note that *δ*^56^Fe values are reported versus igneous rock to allow direct comparison with the data of Severmann *et al.* [54]. On this scale, the *δ*^56^Fe value of the IRMM-14 standard (which is the notation used in the rest of this manuscript) is −0.09‰ [36].

Figure 2*a,b* illustrates the best-fit simulations for the derivation of the effective iron isotope fractionation factors. For the Monterey Canyon (MC) sediment, the model provides a good fit to the measured bulk Fe-mineral distributions and pore-water DFe concentrations (figure 2*a,b*). Modelled DFe concentrations slightly underpredict measured concentrations (figure 2*b*), whereas the modelled benthic flux is approximately 27% higher than measured benthic fluxes from nearby locations (note that the measured fluxes are not from the same location nor the same sampling time as the sediment data) (table 3). Overall, the model is able to capture the major features of MC iron geochemistry, such as the persistence of reactive iron oxides and DFe with depth and a limited accumulation of FeS_2_ (figure 2). For the Santa Barbara Basin (SBB) sediment, the model reproduces the measured Fe-mineral distributions, the depletion of DFe at depth and the benthic DFe flux (figure 2*f,g*, table 3). Although the benthic DFe flux is comparable to the measured flux, the model underestimates the subsurface DFe peak (figure 2*g*). Since this site is hypoxic and has no solute transport via bio-mixing or bio-irrigation, the mismatch reflects either an imbalance in DFe production from FeOOH dissolution and loss of DFe into particulate sulfide or non-steady-state diagenesis. While we are unable to resolve this issue conclusively, we do not find it to be a major source of uncertainty in our derived effective isotope fractionation factors (see below).

**Table 3.** Study sites and comparison of geochemical data with the model output of the sites used for initial model calibration.

**Name**	**latitude longitude**	**water depth (m)**	**bottom-water O_2_ (µM)**		**carbon oxidation rate (mmol m^−2^** **d^−1^)**		**benthic DFe flux (µmol m^−2^** **d^−1^)**		***δ*^56^Fe-DFe of benthic flux (‰)**	
			data	model	data	model	data	model	data	model
Monterey Canyon (MC)	36°47.67′ N 121°53.65′ W	450	>100^a^	280	6–17^a^	7.06	1.1–15^a^	19	−2.7 ± 1.1^d^	−1.3
Santa Barbara Basin (SBB)	34°16.87′ N 119°54.84′ W	496	∼10^b^	10	<4^b^	3.85	>331^c^	248	−3.6 ± 0.7^c^	−2.9

^a^Data from [55]. Note that these fluxes are for a nearby site at a shallower depth and thus not the same site as where the sediment data was collected.

^b^Mineralization rate from [46], based on data from [53], which is from the deepest point of the SBB. Pore-water ammonium and sulfate profiles suggest that the carbon oxidation rate is lower at 496 m depth.

^c^Data for the California margin and Borderland Basins at nearby sites [13].

^d^Data for the Eel River Shelf and Umpqua River Shelf [13], which are comparable sites to the Monterey Canyon site (bioturbated and oxygenated water column). Isotope values are only shown for reference, and the model has not been calibrated to these values.

The best fit *δ*^56^Fe-DFe profile for the Monterey Canyon sediment tracks the measured profile remarkably closely, increasing from a low value of approximately −3.0‰ at the sediment surface and peaking at approximately −1.0‰ at 5 cm depth, followed by a decrease and stabilization at around −1.5‰ (figure 2*d*). Consistent with the measured data, there is very little downcore variation in modelled *δ*^56^Fe-FeHCl (−0.5‰) which includes FeOOH, sorbed Fe^2+^ and FeS, although the model does not reproduce the very low −0.9‰ at the sediment–water interface (figure 2*c*). This does not affect the overall *δ*^56^Fe-DFe pattern that is the focus of this study. The measured *δ*^56^Fe-FeS_2_ profile shows a significant amount of scattering in the upper sedimentary layers, but the general decrease from approximately −0.5‰ near the sediment–water interface (SWI) to approximately −0.8‰ at 50 cm depth is broadly reproduced by the model (figure 2*e*). The concentration of FeS_2_ was very low in the MC sediment (figure 2*e*) and its *δ*^56^Fe-FeS_2_ could have been influenced by a (variable) detrital input, which is not included in our model. These uncertainties directly translate into the effective fractionation factor for pyrite precipitation fitted for the MC sediment, which thus remains uncertain (see below).

For the Santa Barbara Basin, the model reproduces the measured *δ*^56^Fe-DFe data well, being relatively more negative at the sediment surface, and increasing to +1‰ at around 15 cm depth (figure 2*i*). The modelled *δ*^56^Fe-FeHCl profile shows a rapid increase near the SWI (figure 2*h*), driven by the preferential loss of more negative iron isotopes during dissimilatory iron reduction, which are subsequently released to the overlying water column as a benthic flux, consistent with observations (table 3). The modelled *δ*^56^Fe-FeHCl profiles then show a more gradual increase with depth with a slight offset from the measurements of approximately 0.3‰ (figure 2*h*). The modelled *δ*^56^Fe-FeS_2_ profile follows the initial decrease in the measured *δ*^56^Fe-FeS_2_ profile well, but with an increase toward the bottom of the core (figure 2*j*). This mismatch is probably caused by a slight overestimation of the pyrite precipitation rate at depth (figure 2*f*).

Overall, the diagenetic model is able to capture the important trends in bulk concentration and isotopic signatures throughout the sediment column at two very different field sites (figure 2). Furthermore, it simulates the expected magnitude and isotopic composition of the benthic DFe flux (table 3). More importantly, the observed trends were reproduced by applying the same effective isotope fractionation factors that are consistent with literature values (table 4). The effective isotope fractionation factor for FeS_2_ precipitation is an exception since model fitting results in different effective isotope fractionation factors of −0.4‰ for MC and −0.7‰ for SBB (table 4). A value of −0.78 ± 0.15‰ has been previously derived from the same data [54], which is similar to −0.51 ± 0.22‰ obtained in laboratory experiments [83]. In the sensitivity analyses, we apply an effective overall isotope fractionation factor of −0.7‰ for pyrite precipitation and for the other processes the derived effective isotope fractionation factors listed in table 4 are used (electronic supplementary material, Info. §2). A limitation of our approach is that the data available for model calibration/validation is very limited. There are currently no datasets available that include isotope measurements for pore-water and solid-phase concentrations, as well as *in situ* fluxes collected simultaneously. As a result, the effective iron fractionation factors are only calibrated using data from two shallow field sites (table 3) and its applicability to deeper sediments (less than 1 km) or different ocean basins thus remains untested.

### 3.2. Effect of bioturbation on the benthic iron flux and its isotopic signature under different bottom-water redox conditions

The results of the five bioturbation activity scenarios (§2.4), simulated using the idealized model set-up under a range of plausible environmental conditions, highlight the importance of the dynamic interplay between oxygen and macrobenthic activity on benthic iron fluxes and their isotopic signature.

#### 3.2.1. Magnitude of the benthic iron flux

**Figure 3.**
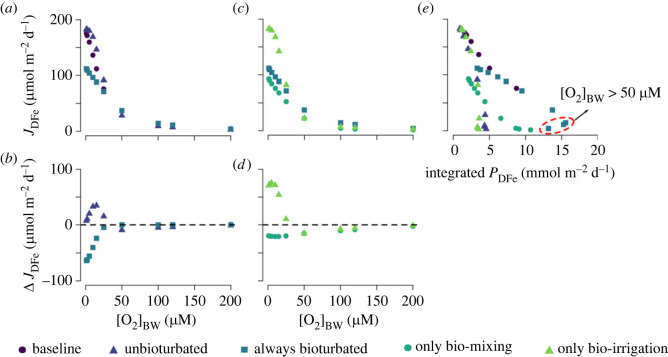
(*a*)–(*d*) Effect of bioturbation on the magnitude of the benthic DFe flux (*J*_DFe_) for different bottom-water oxygen concentrations using the idealized model. Data in panel (*b*) are calculated as the difference between the ‘always bioturbated’ or ‘unbioturbated’ run and the ‘baseline’ run. Data in panel (*d*) are calculated as the difference between the ‘only bio-mixing’ or ‘only bio-irrigation’ run and the ‘always bioturbated’ run. (*e*) Magnitude of the benthic DFe flux plotted against the integrated production rate of DFe (*P*_DFe_), which is a proxy for the number of times Fe is cycled between its oxidized and reduced states in the sediment [24,28]. The red circle indicates the points where [O_2_]_BW_ was higher than 50 µM.

**Figure 4.**
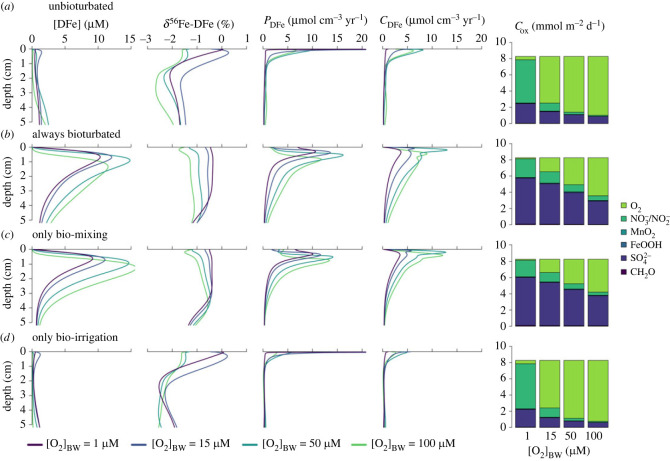
Vertical diagenetic profiles of dissolved iron concentrations ([DFe]), *δ*^56^Fe signature of DFe (*δ*^56^Fe-DFe), total ferrous iron production rate (*P*_DFe_), total ferrous iron consumption rate (*C*_DFe_) for different bottom-water oxygen concentrations, and partitioning of the individual mineralization pathways (note that O_2_, NO_3_^−^ and SO_4_^2−^ are dominant and MnO_2_, FeOOH and CH_2_O are too low to be visible). (*a*) Bioturbation is set to zero. (*b*) Bioturbation is independent of oxygen concentrations and always at its maximum (table 2). (*c*) Only bio-mixing is turned on and independent of oxygen concentrations. (*d*) Only bio-irrigation is turned on and independent of oxygen concentrations.

For unbioturbated sediments, the DFe flux (*J*_DFe_) rapidly decreases from greater than 150 µmol m^−2^ d^−1^ to less than 50 µmol m^−2^ d^−1^ with increasing [O_2_]_BW_ and then becomes essentially zero at [O_2_]_BW_ > 50 µM (figure 3*a*). At low [O_2_]_BW_, the oxygen penetration depth (OPD) is shallow, and most Fe redox cycling is concentrated near the SWI, supporting higher DFe fluxes out of the sediment (figure 4*a*). More importantly, low O_2_ concentrations result in inefficient oxidation of the reduced DFe, thus allowing a significant DFe flux from the sediment (figure 3*a*). An increase in [O_2_]_BW_ moves Fe production and consumption further away from the sediment–water interface, which increases the redox recycling of Fe and provides a more efficient reoxidation barrier for DFe (figure 4*a*). As a consequence, decreasing *J*_DFe_ is observed with an increase in the integrated production rate of DFe (*P*_DFe_) (figure 3*a,e*).

Bioturbated sediments reveal a similar overall decrease in *J*_DFe_ with increasing [O_2_]_BW_ (figure 3*a*). However, the presence of bioturbating fauna attenuates the high DFe fluxes simulated at low [O_2_]_BW_ (less than 50 µM), while it slightly amplifies the very low DFe fluxes simulated for higher [O_2_]_BW_ conditions (figure 3*a,b*). At low [O_2_]_BW_, bio-mixing drives a decrease in *J*_DFe_ relative to the unbioturbated sediment (figure 3*b*). Although bio-mixing directly enhances the porewater concentration of DFe in the sediment by mixing both organic matter and iron oxides deeper down in the sediments, it also stimulates the consumption of DFe via precipitation or reoxidation reactions (figure 4*a,c*) [24,85]. Furthermore, bio-mixing moves Fe cycling away from the SWI, which increases the diffusional distance to the sediment surface and thus allows more efficient reoxidation of reduced Fe, which stimulates iron redox recycling (figure 4*a,c*). The positive effect of bioturbation on *J*_DFe_ fluxes at higher [O_2_]_BW_ is only observed when both bio-mixing and bio-irrigation work in concert. This is because bio-mixing is required to stimulate Fe cycling and build up pore-water DFe concentrations, while bio-irrigation transports DFe out of the sediment (figure 4*c,d*). Hence, while the individual effects of bio-mixing and bio-irrigation affect the sediment biogeochemistry in different ways, both bio-mixing and bio-irrigation contribute to increasing *J*_DFe_ under oxic bottom-water conditions.

#### 3.2.2. Isotopic signature of the benthic iron flux

**Figure 5.**
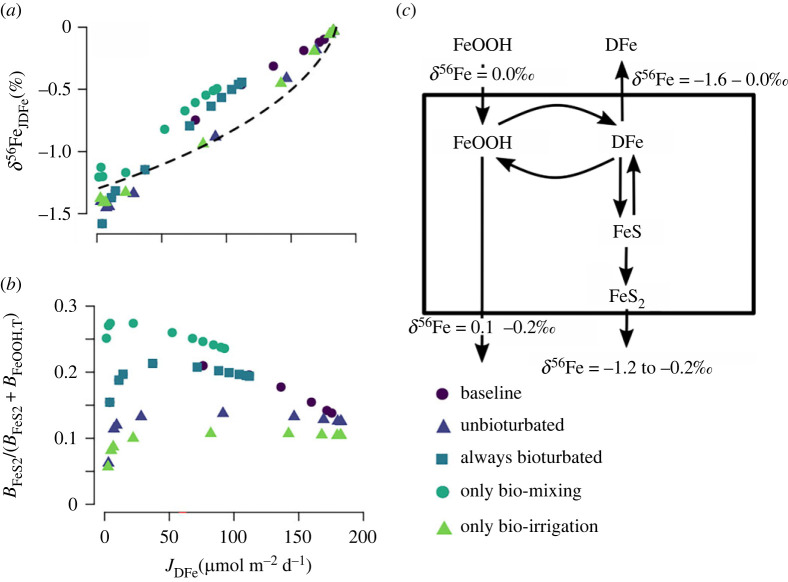
(*a*) *δ*^56^Fe signature of the benthic DFe flux plotted against the benthic DFe flux. The dashed line shows a Rayleigh fractionation model (equation (3.1)). (*b*) Burial flux of pyrite (*B*_FeS2_) relative to the total Fe-mineral burial flux (*B*_FeS2_ + *B*_FeOOH,T_) plotted against the benthic DFe flux. (*c*) Conceptual figure of the modelled iron cycle, with the range of model-predicted isotope signatures for the burial fluxes of FeS_2_ and FeOOH, and the benthic DFe flux.

The isotopic signature of *J*_DFe_ (*δ*^56^Fe_JDFe_) shows a strong positive correlation with *J*_DFe_ (figure 5*a*). The relationship between *δ*^56^Fe_JDFe_ and *J*_DFe_ is partly driven by a Rayleigh distillation effect due to the semi-open nature of aquatic sediments [86,87]. Benthic DFe is derived from the reduction of the finite FeOOH deposition flux and can escape the sediment as a benthic return flux. Hence, as more DFe escapes the sediment through the sediment–water interface, less FeOOH remains. Note that the apparent Rayleigh distillation effect is a consequence of transport out of the sediment, not of the fractionation effect during the reactions (since most reactions involving iron produce equilibrium isotope fractionations). If *δ*^56^Fe_JDFe_ would be solely determined by the apparent Rayleigh distillation effect, we should be able to express the expected *δ*^56^Fe_JDFe_ as a Rayleigh fractionation curve,

3.1 $$\delta^{56}{\mathrm{Fe}}_{\mathrm{JDFe}}=(1000.0+d_{0})\frac{\left(1-{\mathrm{fr}}^{\alpha_{\mathrm{reac}}}\right)}{\left(1-\mathrm{fr}\right)}-1000.0,$$

where *d*_0_ is the isotope signature of the reactant, fr is the remaining fraction of the reactant and *α*_reac_ is the fractionation factor of the reaction. In this case, the reactant is FeOOH, which has an isotopic composition of 0.0‰ (table 2), hence *d*_0_ = 0.0‰. The applied effective fractionation factor of the dissimilatory iron reduction is (*α*_reac_ = *α*_eff,FeOOH−DFe_ =) 0.9987 (table 4, equation (2.4)). The maximum amount of Fe that can leave the sediment as a benthic flux is 170 µmol Fe m^−2^ d^−1^ (figure 3*a*), fr can thus be calculated as (170 – *J*_DFe_). Equation (3.1) assumes that (i) all DFe released from the sediment is derived from FeOOH with an effective fractionation factor of −1.3‰, and (ii) 170 µmol Fe m^−2^ d^−1^ is the maximum amount of DFe that can be released. We can compare the expected *δ*^56^Fe_JDFe_ of the Rayleigh distillation effect (equation (3.1)) with the modelled *δ*^56^Fe_JDFe_ (figure 5*a*) to elucidate the secondary controls on *δ*^56^Fe_JDFe_.

Deviations from the idealized Rayleigh fractionation curve are caused by other removal pathways of Fe from the sediment column, that is, burial of FeOOH and FeS_2_. When Fe is buried in its oxidized form (FeOOH) it has a more positive isotope signature compared with the case where Fe is buried as FeS_2_ (figure 5*c*). Higher burial rates of FeOOH, or lower burial rates of FeS_2_, will thus shift *δ*^56^Fe_JDFe_ to more negative values, and vice versa. At J_DFe_ < 50 µmol Fe m^−2^ d^−1^, the unbioturbated model runs show more negative values than expected. This correlates with the relative importance of FeOOH as a burial phase, which shifts *δ*^56^Fe to more negative values (figure 5*b*). With increasing *J*_DFe_, the relative importance of Fe burial phases in the unbioturbated model runs remains constant, which explains why this experiment more closely follows a typical Rayleigh fractionation (figure 5*a,b*). The baseline and always bioturbated model experiments also plot below the Rayleigh fractionation line at low *J*_DFe_, but shift to more positive than expected values when *J*_DFe_ increases—consistent with an increase in FeS_2_ burial (figure 5*a,b*). This shift to more positive values is also seen in the bio-mixing model run (figure 5*a*), indicating that bio-mixing increases FeS_2_ precipitation and burial by stimulating sulfate reduction, as biomixing introduces more reactive organic carbon into the anoxic part of the sediment (figures 5*b* and 3*c*). Bio-irrigation does not have any strong effect on *δ*^56^Fe_JDFe_ but slightly shifts *δ*^56^Fe_JDFe_ to more negative values when bio-mixing is also active (compare the bio-mixing experiment with the always bioturbated experiment in figure 5*a*). In summary, *δ*^56^Fe_JDFe_ is primarily controlled by the magnitude of the benthic Fe flux, while the bio-mixing component of bioturbation shifts *δ*^56^Fe_JDFe_ to higher values by stimulating the burial of FeS_2_.

### 3.3. Predictive functions of the isotopic composition of benthic iron fluxes

In this section, we use the idealized model set-up to derive predictive functions based on the most important drivers of the benthic DFe flux and its isotopic signature: bioturbation, [O_2_]_BW_, Cox and J_FeOOH,T_. We do this for a modern seafloor with bioturbation (bioturbated seafloor) and a seafloor without bioturbation (unbioturbated seafloor).

#### 3.3.1. Bioturbated seafloor

**Figure 6.**
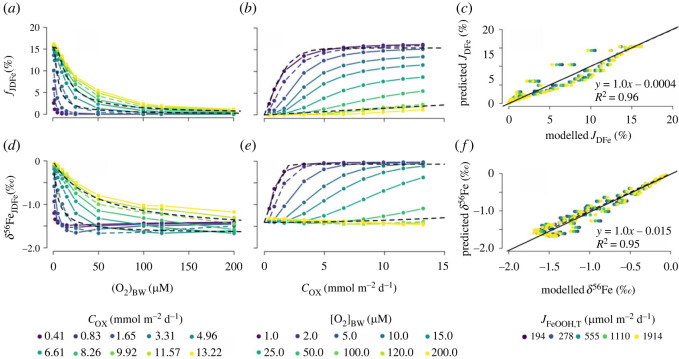
Simulated benthic DFe flux relative to the FeOOH influx (*f*_JDFe_ = |*J*_DFe_/_JFeOOH,T_|) and the *δ*^56^Fe signature of the DFe flux (*δ*^56^Fe_JDFe_) relative to (*a*),(*d*) bottom-water oxygen concentrations ([O_2_]_BW_), (*b*),(*e*) carbon oxidation rate (*C*_ox_) for a modern seafloor. In panels (*a*) and (*d*) the results for *C*_ox_ = 3.31 mmol m^−2^ d^−1^ and *C*_ox_ = 9.92 mmol m^−2^ d^−1^ (dashed coloured lines) are compared with equation (1.1) and (3.2) (dashed black lines). In panels (*b*) and (*e*), the results for [O_2_]_BW_ = 2 µM and [O_2_]_BW_ = 100 µM (dashed coloured lines) are compared with equations (1.1) and (3.2) (dashed black lines). Panels (*c*) and (*f*) show the correlation between the modelled (*c*) benthic DFe flux (*J*_DFe_) and (*f*) *δ*^56^Fe, and the values predicted using the empirical functions for different FeOOH influxes (*J*_FeOOH,T_) (see main text).

We build on the study of Dale *et al.* [9], who derived a transfer function to quantify *J*_DFe_ as a function of Cox, *J*_FeOOH,T_ and [O_2_]_BW_ (equation (1.1)). We repeat the same experiment (varying [O_2_]_BW_ from 1 to 200 µM and Cox from 0.4 and 13.2 mmol m^−2^ d^−1^; table 2), to derive a similar predictive function for the *δ*^56^Fe value of *J*_DFe_ (*δ*^56^Fe_JDFe_). Model results indicate that *δ*^56^Fe_JDFe_ behaves similarly to *J*_DFe_ (figure 6*d,e*) and the transfer function for *δ*^56^Fe_JDFe_ is best described as

3.2 $$\delta^{56}{\mathrm{Fe}}_{\mathrm{JDFe}}=\frac{1.65({\mathrm{Cox}}^{2}/{\left[O_{2}\right]}_{\mathrm{BW}})}{2.09+({\mathrm{Cox}}^{2}/{\left[O_{2}\right]}_{\mathrm{BW}})}-1.67,$$

where Cox is in mmol m^−2^ d^−1^, [O_2_]_BW_ is in µM and *δ*^56^Fe_JDFe_ is in ‰. This function is independent of J_FeOOH,T_ and explains 95% of the variance in the modelled isotope values (figure 6*f*). The maximum expressed effective overall fractionation, relative to the *δ*^56^Fe of the FeOOH entering the sediment, is −1.67‰ for the tested ranges of Cox and [O_2_]_BW_. For instance, if the FeOOH deposited on the seafloor has a *δ*^56^Fe of −1.0‰, the minimum value of *δ*^56^Fe of the dissolved iron flux will be −2.67‰. The amount of iron oxides delivered to the sediment (*J*_FeOOH,T_) has a small effect on the model output (figure 6*c,f*), but negligible compared with Cox and [O_2_]_BW_.

**Figure 7.**
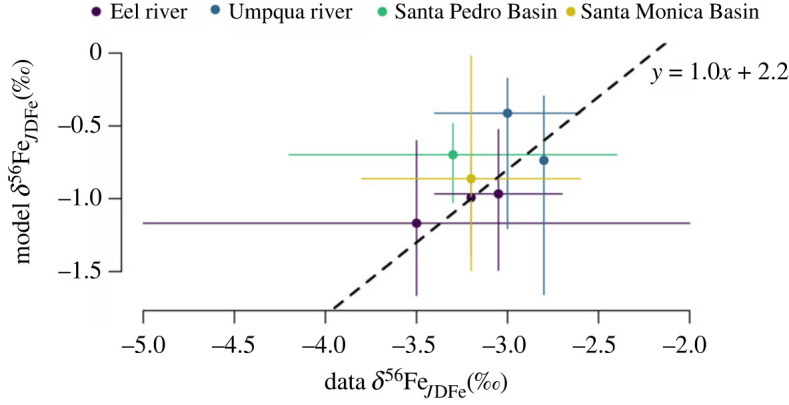
Modelled ^56^Fe isotope signature of the benthic iron flux (*δ*^56^Fe_JDFe_) versus *in situ* data from [13,38]. Solid lines represent the uncertainty in measured *δ*^56^Fe_JDFe_ (*x*-axis), or uncertainty in Cox or [O_2_]_BW_ (*y*-axis). The trendline is illustrative to show the ideal 1 : 1 correlation with an offset of 2.2‰ and does not represent a statistical correlation.

**Figure 8.**
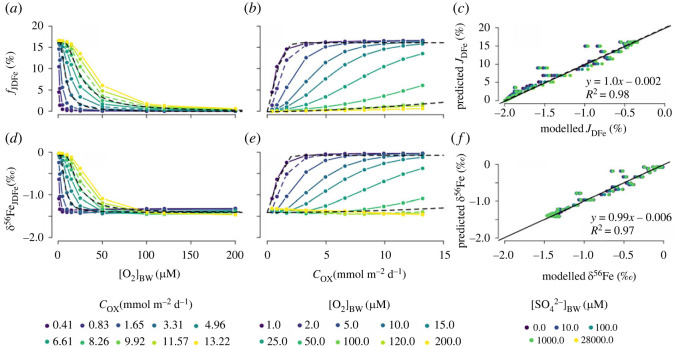
Simulated benthic DFe flux relative to the FeOOH influx (*f*_JDFe_ = |*J*_DFe_/_JFeOOH,T_|) and the *δ*^56^Fe signature of the DFe flux (*δ*^56^Fe_JDFe_) relative to (*a*),(*d*) bottom-water oxygen concentrations ([O_2_]_BW_), (*b*),(*e*) carbon oxidation rate (*C*_ox_) for a Precambrian seafloor. The dashed black line in panels (*a*),(*b*),(*d*),(*e*) are the proposed functions for the magnitude (*a*),(*b*) and the *δ*^56^Fe signature (*d*),(*e*) of the benthic DFe flux. In panels (*a*),(*d*) the results for *C*_ox_ = 3.31 mmol m^−2^ d^−1^ and *C*_ox_ = 9.92 mmol m^−2^ d^−1^ (dashed coloured lines) are compared with the new functions in equations (3.3) and (3.4). In panels (*b*),(*e*) the results for [O_2_]_BW_ = 2 µM and [O_2_]_BW_ = 100 µM (dashed coloured lines) are compared with the new functions in equations (3.3) and (3.4) (dashed black lines). Panels (*c*) and (*f*) show the correlation between the modelled (*c*) benthic DFe flux (*J*_DFe_) and (*f*) *δ*^56^Fe, and the values predicted using the empirical functions for different FeOOH influxes (*J*_FeOOH,T_) (see main text).

Due to a lack of available empirical data of benthic iron fluxes and their isotopic composition, it is difficult to validate the proposed transfer function (equation (3.2)) on a global scale. Furthermore, the *δ*^56^Fe of the incoming FeOOH is generally unknown. However, we can qualitatively compare our model predictions with available site-specific data. If we assume that the *δ*^56^Fe signature of the incoming FeOOH is identical between the coastal sites studied in [13] (they are all located along the California and Oregon continental margin), the predicted *δ*^56^Fe_JDFe_ should be linearly related to the measured *δ*^56^Fe_JDFe_, with a constant offset (which is the *δ*^56^Fe value of the incoming FeOOH, which we here set at 2.2‰). Indeed, there is a broad positive relationship between predicted and measured *δ*^56^Fe_JDFe_ (figure 8). While more data is needed to validate equation (3.2) on a global scale, the positive relationship between predicted and measured values brings some degree of confidence to our model predictions (figure 7).

#### 3.3.2. Unbioturbated seafloor

Given that *J*_FeOOH,T_ shows little impact on the model output of our previous experiments for the modern seafloor (figure 6*c,f*), we do not repeat here the results of varying *J*_FeOOH,T_ for an unbioturbated seafloor. Instead, we focus on the impact of ${\left[{\mathrm{SO}}_{4}^{2-}\right]}_{\mathrm{BW}}$ concentrations which varied from a few millimolar to 28 mM during the Quaternary [57]. Sulfate concentrations could exert an important control on *J*_DFe_ because the major benthic sink for iron in sediments is the precipitation and burial of pyrite [88].

The global responses of *J*_DFe_ and *δ*^56^Fe_JDFe_ are broadly comparable to the modern bioturbated situation although, as discussed in §3.2, *J*_DFe_ is 30–40% lower at higher [O_2_]_BW_ compared with bioturbated sediments (figure 8*a,b*). We propose an asymptotic function to describe this behaviour,

3.3 $$J_{\mathrm{DFe}}=(0.161-0.161e^{-3.67(\mathrm{Cox}/{\left[O_{2}\right]}_{\mathrm{BW}})})J_{\mathrm{FeOOH},T},$$

where Cox is in mmol m^−2^ d^−1^, [O_2_]_BW_ is in µM, and *J*_FeOOH,T_ and *J*_DFe_ are in µmol m^−2^ d^−1^. This function explains 98% of the variance in the modelled fluxes (figure 8*c*). Surprisingly, decreasing ${\left[{\mathrm{SO}}_{4}^{2-}\right]}_{\mathrm{BW}}$ exerts a negligible impact on simulated *J*_DFe_ (figure 8*c*). This suggests that, in unbioturbated sediments, reoxidation of DFe in the oxic zone is more important than the trapping of DFe as iron-sulfide minerals. We observe a slight increase in *J*_DFe_ at higher ${\left[{\mathrm{SO}}_{4}^{2-}\right]}_{\mathrm{BW}}$ (points shift to the right in figure 8*c*). This occurs because some oxidized iron minerals are not reactive towards organic matter, but can be reduced by dissolved sulfide [45,89]. By increasing sulfate concentrations, iron reduction is promoted via the sulfide intermediate, which leads to a slight increase in modelled *J*_DFe_. In the absence of sulfide, some of the iron oxides could be reduced by oxidation of methane, although studies suggest that methane is not efficient at reducing iron compared with sulfide [90]. The insensitivity of the benthic iron flux to sulfate availability also shows that adding iron carbonate (siderite) or iron phosphate (vivianite) precipitation, which could be an important DFe sink in sulfide-poor conditions, would have had no impact on model results compared with the sulfide-rich scenario.

As expected, the trends in *δ*^56^Fe_JDFe_ relative to Cox and [O_2_]_BW_ behave very similarly as *J*_DFe_ (figure 8*d,e*), and consequently, the predictive function for the *δ*^56^Fe_JDFe_ resembles equation (3.3)

3.4 $$\delta^{56}{\mathrm{Fe}}_{\mathrm{JDFe}}=(1.60-1.34e^{-3.67{\left(\mathrm{Cox}/{\left[O_{2}\right]}_{\mathrm{BW}}\right)}^{2}})-1.67,$$

where Cox is in mmol m^−2^ d^−1^, [O_2_]_BW_ is in µM, and *δ*^56^Fe_JDFe_ is in ‰. This function explains 97% of the variance in the modelled fluxes (figure 8*f*). Note that for [O_2_]_BW_ > 100 µM, *δ*^56^Fe_JDFe_ is essentially invariant, which implies that [O_2_]_BW_ alone is a poor predictor for *δ*^56^Fe_JDFe_.

### 3.4. Importance of bioturbation for the global iron cycle

**Figure 9.**
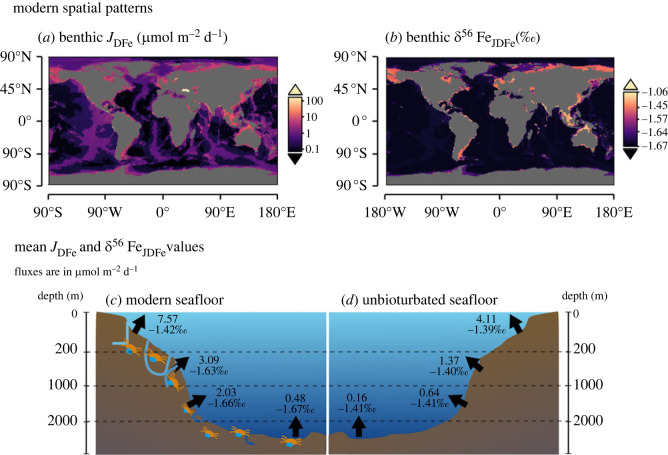
Top row: modern spatial patterns of (*a*) benthic DFe fluxes (*J*_DFe_) and (*b*) the *δ*^56^Fe signature of the benthic DFe flux (*δ*^56^Fe_JDFe_). (*c*) Mean *J*_DFe_ (upper numbers) and *δ*^56^Fe_JDFe_ (lower numbers) per depth interval for (*c*) the modern (bioturbated) seafloor and (*d*) and unbioturbated seafloor. The results in (*a*) were calculated with equation (1.1), and those in (*b*) with equation (3.2). The results in (*c*) were calculated with equations (1.1) and (3.2), and those (*d*) were calculated equations (3.3) and (3.4).

The predictive functions derived in §3.3 allow the assessment of the influence of bioturbation on benthic DFe release and its *δ*^56^Fe signature on the global scale (§2.5). We calculate the mean and total DFe flux for several water depth intervals, as well as the mean *δ*^56^Fe signature of the DFe flux (figure 9, table 5). Interestingly, even though bioturbation only has a small impact on the benthic DFe flux at higher oxygen concentrations at first sight (figure 3), this small local difference amounts to a large difference on a global scale (table 5). Dissolved Fe fluxes for an unbioturbated seafloor (global total: 70 G mol Fe yr^−1^) are much lower than for the modern seafloor (global total: 158 G mol Fe yr^−1^) (table 5). The mean unbioturbated *J*_DFe_ is around 1/3 of the mean bioturbated *J*_DFe_ in the deeper regions of the oceans (figure 9*c,d*), where high oxygen concentrations and lower organic carbon oxidation rates prevent the diffusional release of DFe. In the shallower shelf regions, the mean *J*_DFe_ is still 1.8 times higher in bioturbated conditions (figure 9*c,d*). Overall, global benthic DFe release for an unbioturbated seafloor is less than 50% of the global benthic DFe flux of the modern seafloor (table 5), which suggests that benthic fauna is an essential part of the modern global marine iron cycle, and could be an important Fe source in Fe-limited regions.

**Table 5.** Total dissolved iron fluxes from marine sediments for a modern seafloor (calculated using equation (1.1)) and for an unbioturbated seafloor without bioturbation (calculated using equation (3.3)).

	area^a^ (10^12^ m^2^)	mean Cox^b^ (mmol m^−2^ d^−1^)	total DFe flux^c^ (Gmol yr^−1^)	
			modern	unbioturbated
shelf (0–200 m)	27.12	9.4	75 ± 38	41 ± 20
upper slope (200–1000 m)	16.01	3.0	18 ± 9	8.0 ± 4.0
lower slope (1000–2000 m)	15.84	1.5	12 ± 6	3.7 ± 1.8
deep-sea (> 2000 m)	302.5	0.4	53 ± 26	17 ± 8.5
total			**158** **±** **47**	**70** ± **22**

^a^[49].

^b^[52].

^c^Relative error on the benthic Fe flux, calculated using equations (1.1) and (3.3), was estimated at 50% by [9], based on the uncertainty in sedimentary Fe contents [66].

Additionally, bioturbation increases the range in *δ*^56^Fe values from DFe released from the seafloor (figure 9*c,d*). Our model experiments show that *δ*^56^Fe_JDFe_ values are near approximately −1.41‰ for [O_2_]_BW_ > 50 µM, and only show some variability below that oxygen concentration (figure 8*d*). By contrast, with bioturbation, *δ*^56^Fe_JDFe_ values show significant variability at all oxygen concentrations (figure 6*d*). Regardless of the range, both bioturbated and unbioturbated scenarios show similar spatial trends. More negative *δ*^56^Fe values are found in the deep sea, where *J*_DFe_ is lower, whereas more positive *δ*^56^Fe values are found near shore, where *J*_DFe_ is higher (figure 9*b–d*; table 5). Overall, the *δ*^56^Fe signatures of *J*_DFe_ are consistently more negative (up to approx. 0.3‰) in the modern seafloor (figure 9*c,d*).

Note that our predictions for the deeper part of the ocean (greater than 1000 m water depth) could be biased by our assumption of fixed organic matter reactivity (§2.4). Nevertheless, the estimated benthic flux for deep-sea sediments is less than 0.5–2.0 µmol DFe m^−2^ d^−1^ (figure 9), which is in the range of DFe fluxes estimated from non-reductive dissolution of FeOOH [38]—a potentially important DFe source in these low-productive sediments. This would impact the *δ*^56^Fe signature of the benthic DFe flux (which is approx. 0‰ for non-reductive dissolution), although the low flux magnitude means the impact on the oceanic *δ*^56^Fe is expected to be small. In addition, the derived transfer functions are based on depth-integrated degradation rates Cox and thus implicitly account for changes in OM flux and/or reactivity.

Our results suggest that the evolution of benthic fauna and the advent of bioturbation around the Ediacaran–Cambrian transition could have significantly altered the oceanic iron cycle. In a world without benthic fauna, reactive dissolved and particulate iron delivered from land would be recycled very inefficiently from the seafloor. This would lead to an accumulation of iron minerals in nearshore and riverine sediments, and low amounts of reactive iron in more offshore sediments. With the advent of bioturbation, sediment mixing and burrow flushing by benthic fauna would have increased the release of DFe from the seafloor, thereby stimulating Fe cycling in the water column and potentially increasing the residence time of Fe in the ocean. At the same time, reactive iron would begin to accumulate in deeper, more offshore sediments. We speculate that this could potentially be observed in the rock record as an increase in reactive iron to total iron ratios in shelf sediments when moving from the late Proterozoic to early Phanerozoic, as sedimentary recycling of iron has been shown to increase its reactivity [91]. Our work adds to a growing body of literature that suggests that the burrowing revolution around the Ediacaran–Cambrian transition had a major impact on the global cycling of sulfur, carbon, phosphorus and oxygen [92–97]. Bioturbation has been suggested to increase phosphorus burial in marine sediments (although this is debatable; [98,99]), which could have limited primary productivity in the early Cambrian, consequently leading to lower atmospheric oxygen concentrations [92,96,97]. Our results suggest that the impact of bioturbation on the Fe cycle could have had the opposite effect. By increasing DFe release from the sediment, bioturbation could have relaxed iron limitation, potentially stimulating primary productivity in Fe-limited regions of the ocean.

## 4. Summary and outlook

In this study, we assessed the influence of bioturbation on benthic dissolved iron (DFe) fluxes and their isotopic signature using reaction-transport modelling. We find that depending on the boundary conditions (bottom-water O_2_ concentrations, carbon oxidation rate, presence/absence of bioturbation) the expressed overall fractionation of the benthic iron flux relative to the *δ*^56^Fe of the FeOOH entering the sediment, can range from −1.67‰ to 0.0‰. Our calibrated effective overall fractionation factors for iron reduction (−1.3‰), iron oxidation (+0.4‰), iron sulfide precipitation (+0.5‰) and dissolution (−0.5‰) and pyrite precipitation (−0.7‰) were fully consistent with experimentally derived values. This suggests that the calibrated effective overall fractionations are robust, but the lack of available field data indicates that more isotopic measurements of pore-water Fe, solid-phase Fe and benthic Fe fluxes from different depositional environments are needed. Future model development should also target more realistic descriptions of iron fractionation in marine sediments.

We found that the influence of bioturbation on DFe fluxes depends on the redox state of bottom waters. Bio-mixing reduces benthic DFe release and gives it a more negative isotopic signature at low bottom-water oxygen concentrations (less than 50 µM), whereas the combination of bio-mixing and bio-irrigation increases benthic DFe release and gives it a more positive isotopic signature burial at higher bottom-water oxygen concentrations (greater than 50 µM) (by stimulating FeS_2_ burial). Globally, bioturbation more than doubles the global benthic DFe flux (from 70 to 158 G mol yr^−1^) and decreases its isotopic signature. Our results emphasize the global importance of bioturbating fauna as ecosystems engineers and should inspire future research on the impact of environmental change on the global iron cycle.

The predictive functions developed here can easily be applied to models of the modern and past oceanic Fe cycle and help advance our understanding of the marine iron cycle. More specifically, coupling our function to a pelagic iron model could predict spatial isotope patterns of dissolved and mineral Fe phases. This would be of major importance for the interpretation of Fe isotope patterns in the geological record, by explicitly accounting for close benthic-pelagic coupling where iron released from the seafloor is reoxidized in the water column and rains back down on the sediment—such as during iron shelf-to-basin shuttling in low-oxygen oceans [100,101].

## Data Availability

The code for the diagenetic model used in this paper is tagged as v. 0.1.0 and is available at: https://doi.org/10.5281/zenodo.4953500 [102]. The code is hosted on GitHub and can be obtained by cloning https://github.com/sevdevel/DiageneticIronIsotopeModel and then checking out the specific release ‘git checkout v0.1.0’. All source code and simulation files required to reproduce the model results presented in this manuscript are stored in the main directory; details are given in the ‘readme.txt’ file in the main directory. The data are provided in electronic supplementary material [103].
